# Granulocyte colony-stimulating factor-producing squamous cell carcinoma of the lower gingiva: a case report

**DOI:** 10.1186/1758-3284-4-35

**Published:** 2012-06-19

**Authors:** Jun-ichi Kobayashi, Akihiro Miyazaki, Takashi Yamamot, Kenji Nakamori, Rina Suzuki, Takeshi Kaneko, Naohiro Suzuki, Hiroyoshi Hiratsuka

**Affiliations:** 1 Department of Oral Surgery, Sapporo Medical University School of Medicine, South 1,West 16, Chuo-ku Sapporo, 060-8543, Japan

**Keywords:** SCC, Oral, G-CSF, Multiple metastasis

## Abstract

The present study summarizes our experience in treating a patient with a suspected granulocyte colony-stimulating factor (G-CSF)-producing squamous cell carcinoma (SCC) of the lower gingiva, which is a rather rare entity.

A 56-year-old woman underwent surgical excision of palate leukoplakia in 1996. In 2009, however, a leukoplakic superficial tumor was detected in the lower left gingiva, for which the patient underwent gingivectomy. This was subsequently diagnosed as SCC. The patient also underwent superselective arterial injection chemotherapy combined with radiotherapy, after local recurrence was observed. The patient was subsequently found to have bone metastasis. After chemotherapy combined with radiotherapy, the patient underwent segmental resection of the lower left jaw, left supraomohyoid neck dissection, and lower jaw reconstruction using titanium plates. Resection of the left femoral tumor and left total knee replacement were also performed. Computed tomography scan performed 1 month after the surgeries revealed multiple lung, liver, spine, and subcutaneous metastases. The patient also exhibited a sudden increase in her white blood cell (WBC) count and a fever that could not be alleviated, despite treatment with antibacterial drugs. A G-CSF-producing tumor was therefore suspected. Serum G-CSF level was high at 250 pg/ml.

The patient's WBC count increased to 32 × 10^3^/ml and her general condition suddenly deteriorated, and she died as a result of multiple organ failure. A final diagnosis of G-CSF-producing SCC of the lower gingiva was made based on the patient's clinical course.

## Background

Solid malignancies are sometimes accompanied by leukocytosis and other leukemoid symptoms, the mechanism of which is attributed to the production of granulocyte colony-stimulating factor (G-CSF) by the tumor cells themselves [1,2]. The ratio at which the malignant tumor accuracy is defined with accessory symptoms such as leukocytosis is not yet clear. G-CSF-producing tumors exhibit significant hyperplastic and metastatic properties and have a very poor prognosis [3]. The present study describes our experience in treating a patient who was thought to have a G-CSF-producing squamous cell carcinoma (SCC) of the lower gingiva, after she developed systemic metastases along with fever and a sudden increase in the white blood cell (WBC) count.

## Case presentation

The patient was a woman in her 56 years old who had initially consulted our hospital in November 1996, at the onset of leukoplakia. The patient’s major complaint was the appearance of leukoplakic lesions corresponding to left side first and second molar equivalent to palate gingiva. The patient had been previously diagnosed with uterine polyps. Her family history was unremarkable. The patient was examined at our department in late November 1996, following a referral from her local dentist, who detected white lesions on the left palate during dental treatment in mid-November of the same year. The patient was diagnosed with leukoplakia, and the palatal leukoplakia was resected through an excisional biopsy in December of the same year. The patient was subsequently followed up on an outpatient basis. In May 2006, white lesions were observed on the left buccal mucosa and the patient was diagnosed with dysplasia. The condition was treated with another surgical resection, and the patient was followed up as an outpatient. In December 2008, a leukoplakic superficial tumor appeared at left side lower canine, the first premolar equivalency department buccal marginal gingiva.

**Figure 1:**
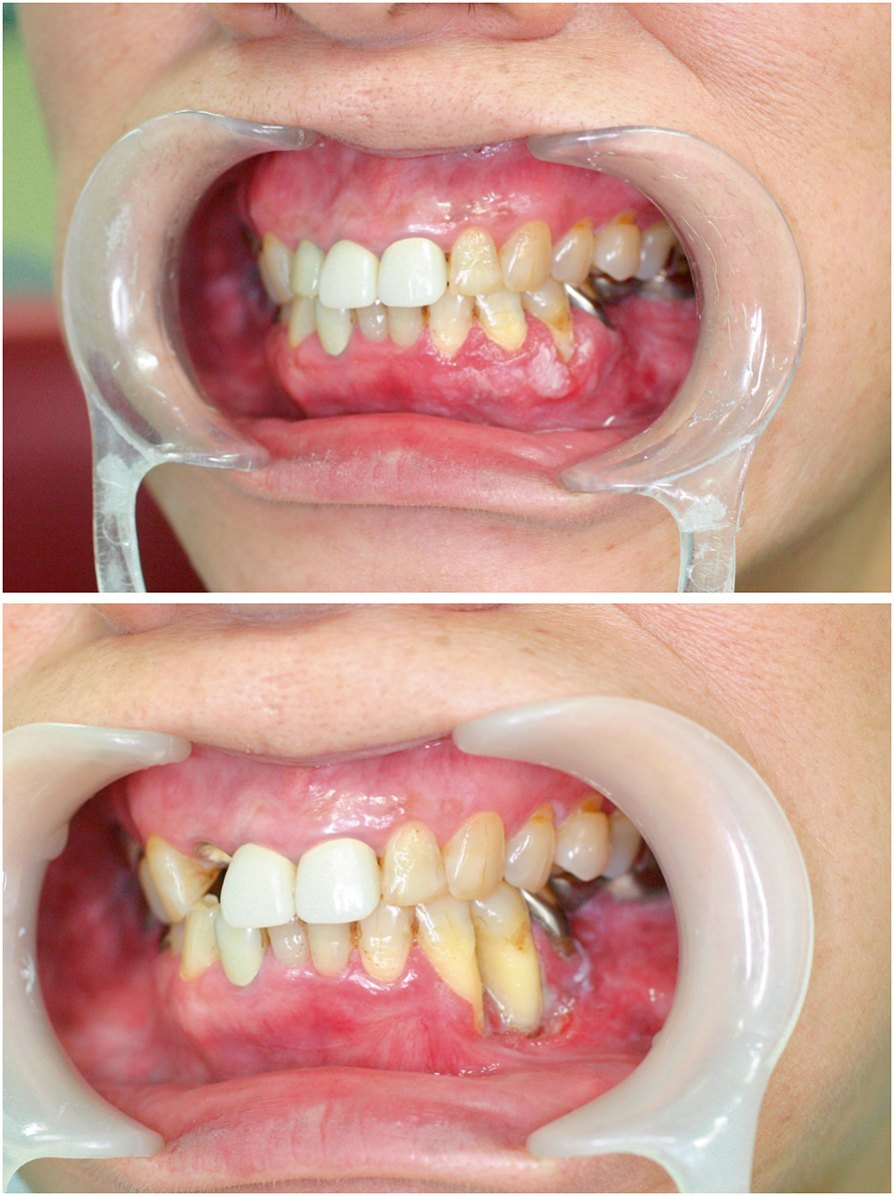
(**Upper) Intraoral photo at initial consultation: leukoderma and erosion were observed at left side lower canine, the first premolar equivalent to palate of gingiva (Lower) Intraoral photo at relapse: hemorrhagic leukoderma and erosion were observed at left side lower canine, the first premolar equivalent to palate of gingiva.**

The patient had moderate physique and was well-nourished, and no swelling of the cervical lymph nodes was observed. Leukoderma and erosion were observed at left side lower canine, the first premolar equivalency department buccal marginal gingiva. (Figure 1 upper).

Panoramic radiography findings did not reveal any resorption of the lower jaw. Computed tomography (CT) findings did not indicate any clear destruction of the lower left jaw or lymph node enlargement, which could be suggestive of metastasis to the neck.

**Figure 2:**
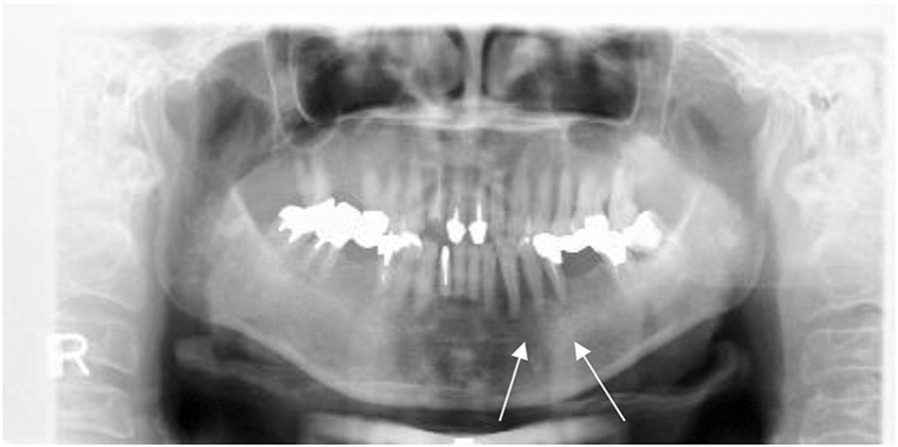
**Panoramic radiograph at relapse.** Bone absorption observed at left side lower canine, the first premolar equivalent to department gingiva.

**Figure 3:**
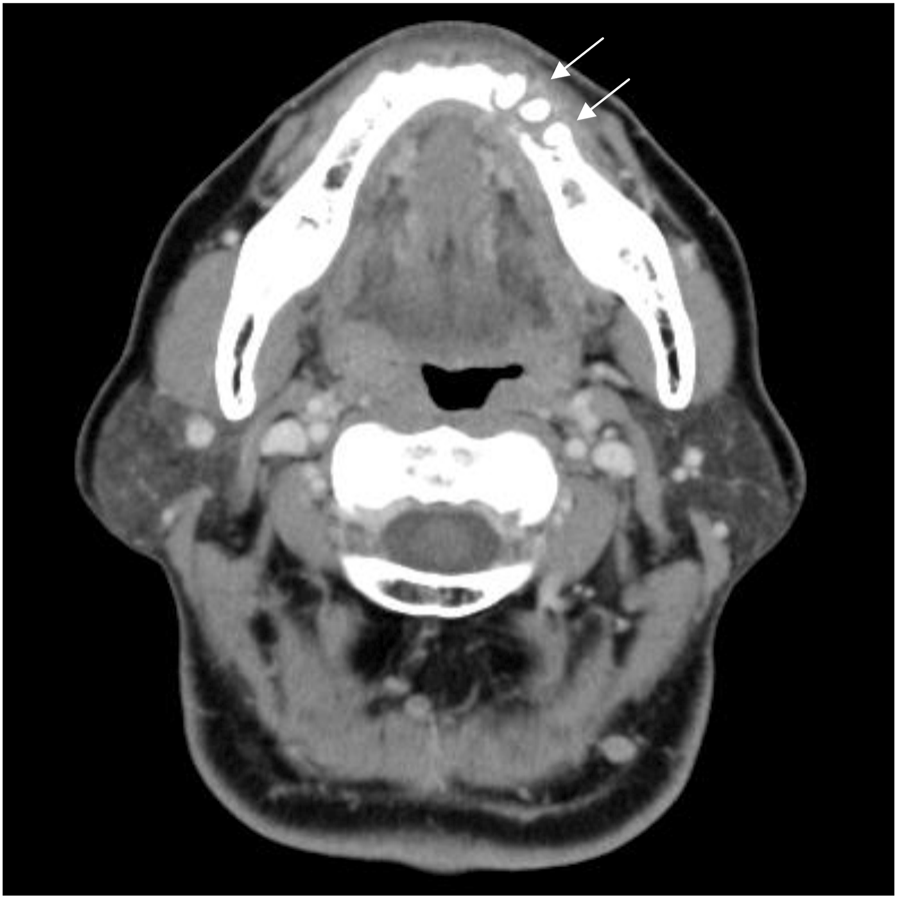
**CT scan at relapse.** Osteolysis observed at left side lower canine, the first premolar, the second premolar equivalent to department gingiva.

The clinical diagnosis for the patient was tumor of lower left gingiva. In January 2009, the patient underwent an excisional biopsy, with resection of the lower left gingiva tumor. Pathological analysis revealed well-differentiated SCC of more than 20 mm (pT2). No swelling of the cervical lymph nodes was observed. Hemorrhagic leukoderma and erosion were observed at left side lower canine, the first premolar equivalent to palate gingiva. (Figure 1 lower). Panoramic radiography revealed resorption of the lower jaw (Figure 2), and CT showed clear destruction of the lower left jaw, but without lymph node enlargement, which could be suggestive of metastasis to the neck (Figure 3).

**Figure 4:**
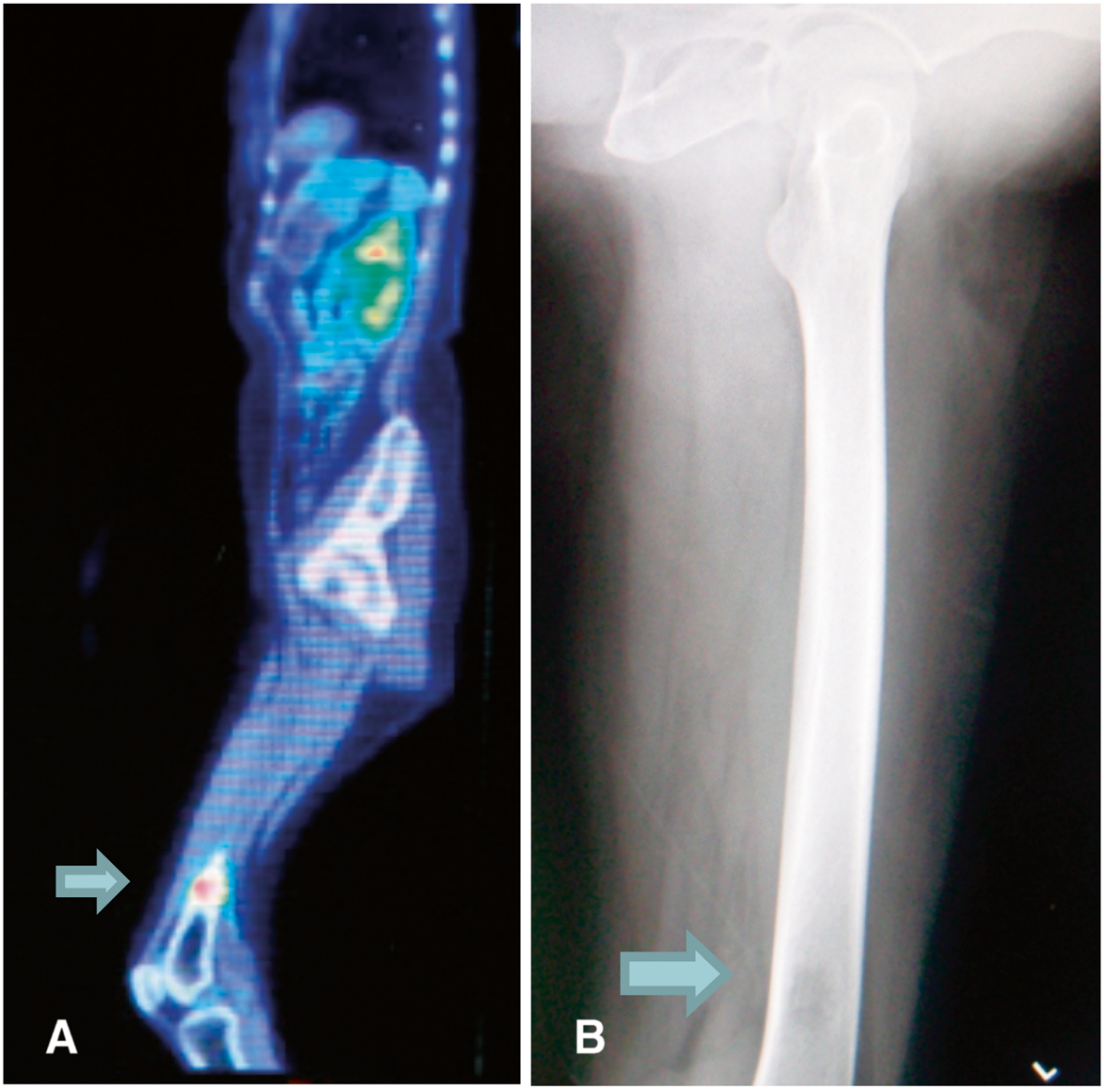
(**A) Flurodeoxyglucose (FDG)-PET/CT scan showed a dense accumulation in the left femur suggestive of tumor metastasis (B) Radiographic image of the left femur revealed bone absorption indicative of metastasis.**

**Figure 5:**
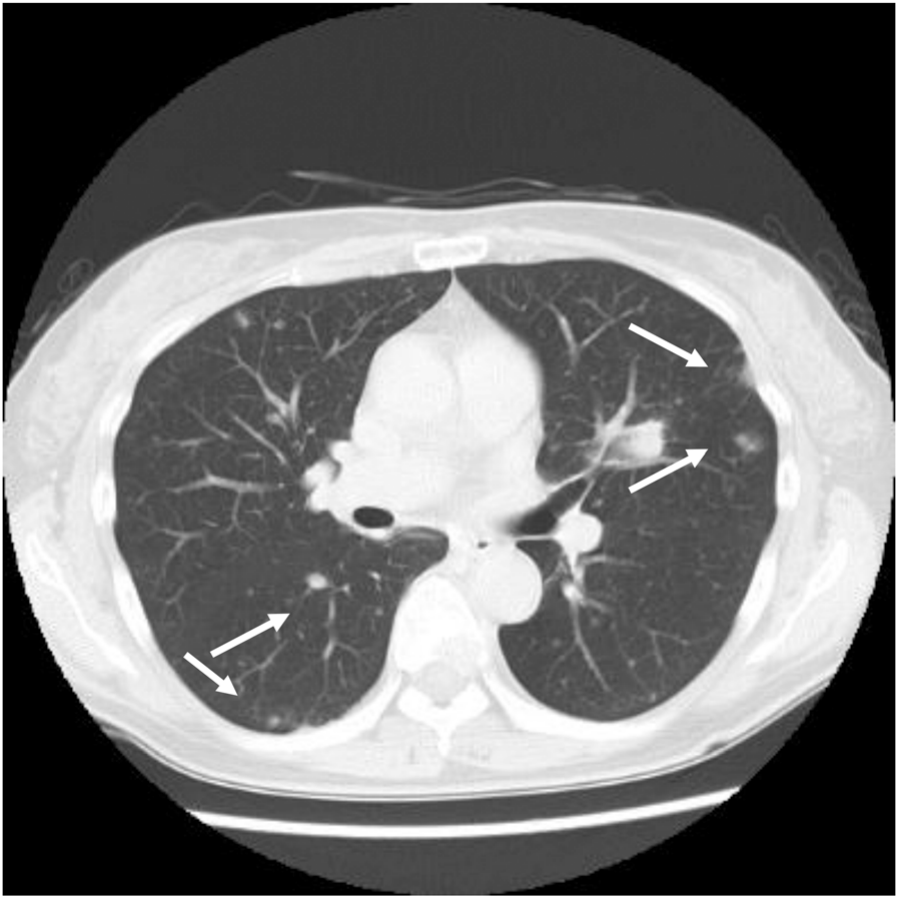
CT findings showed multiple metastases to the lungs.

**Figure 6:**
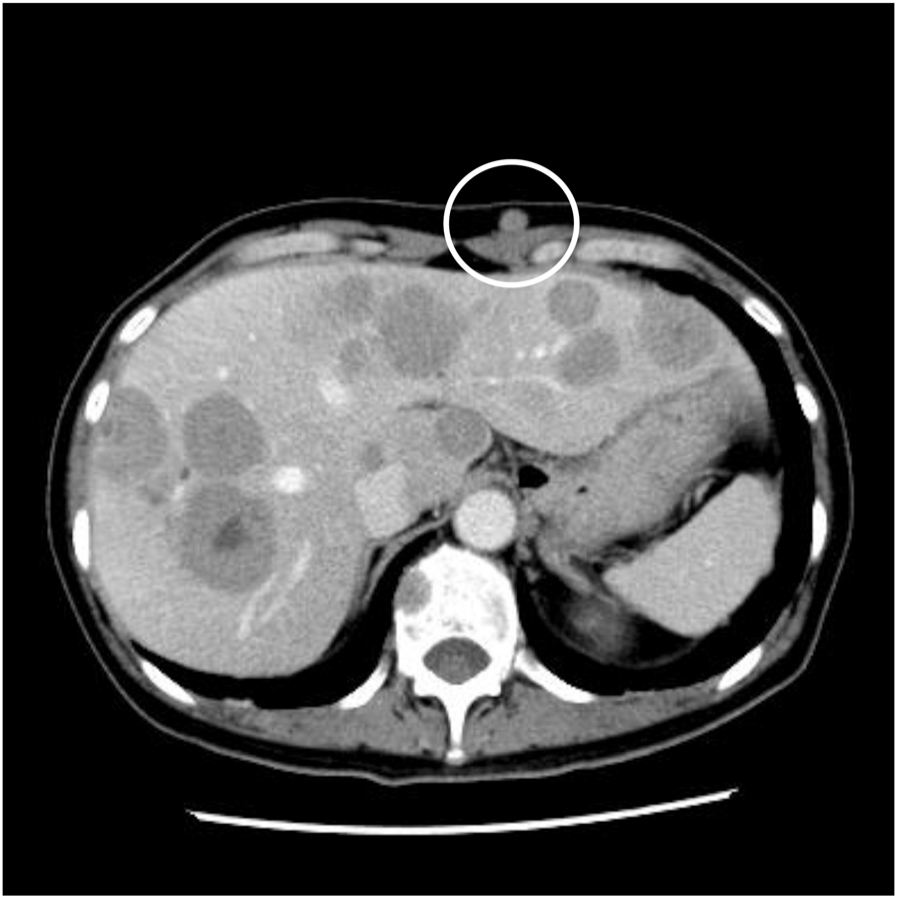
CT findings showed metastases to the liver, spine, mediastinal lymph nodes, and abdominal subcutaneous tissue (indicated by the red circle).

**Figure 7:**
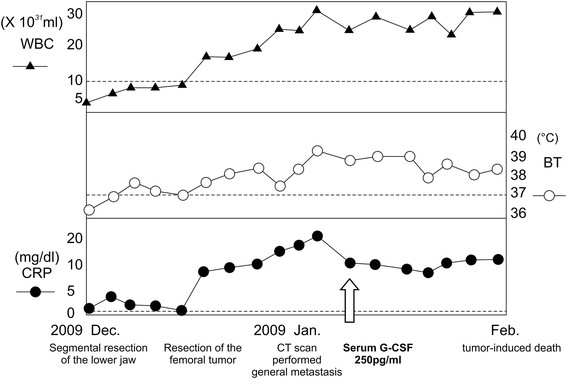
Shift in WBC count, CRP, and body temperature.

On relapse, the patient was clinically diagnosed with a lower left gingiva tumor. The tumor recurred in the lower left gingiva (rT4aN0M0) in July 2009. Systemic chemotherapy was performed with 15 mg bleomycin (BLM). Beginning in August 2009, the patient underwent selective arterial injection chemotherapy, combined with radiotherapy. The chemotherapy regimen consisted of 100 mg cisplatin (CDDP), administered in 4 doses: 30 mg to the lingual artery, 40 mg to the facial artery, 10 mg to the inferior alveolar artery, and 20 mg to the occipital artery. The radiotherapy regimen using Linac consisted of a total of 64 Gray (Gy) irradiation, administered bilaterally in 32 fractions, including levels I and IIa. The patient then complained of pain in the left femur. An imaging study indicated that the tumor had metastasized to the left femur (Figure 4A and 4B); therefore, 300 mg/day of tegafur-uracil (UFT) in combination with radiotherapy, consisting of bilateral irradiation of 45 Gy in 15 fractions, was applied to the metastasis. At the beginning of November 2009, the patient underwent systemic chemotherapy with 135 mg nedaplatin (CDGP) and 90 mg docetaxel (DTX), followed by 300 mg/day UFT at the end of the same month. At the beginning of December 2009, the patient underwent segmental resection of the lower left jaw, left supraomohyoid neck dissection, and lower jaw reconstruction using titanium plates. At the end of December in the same year, the femoral tumor was surgically removed and a total knee replacement was performed. CT performed 1 month after surgery revealed multiple metastases to the lungs (Figure 5), liver, spine, mediastinal lymph nodes, and abdominal subcutaneous tissue (Figure 6). The patient simultaneously exhibited fever and a sudden increase in her WBC count and C-reactive protein (CRP), which failed to abate, despite treatment with the antibacterial drug imipenem hydrate-cilastatin sodium. A G-CSF-producing tumor was suspected; the serum G-CSF level was high at 250 pg/ml (Figure 7). The patient's WBC count eventually increased to 32 × 10^3^/ml. Her general condition suddenly worsened, resulting in death due to multiple organ failure in February 2010; a month since the G-CSF producing tumor was diagnosed. Patient survived for 13 months from her time of tumor diagnosis till her death and her period of survival was uneventful.

**Figure 8:**
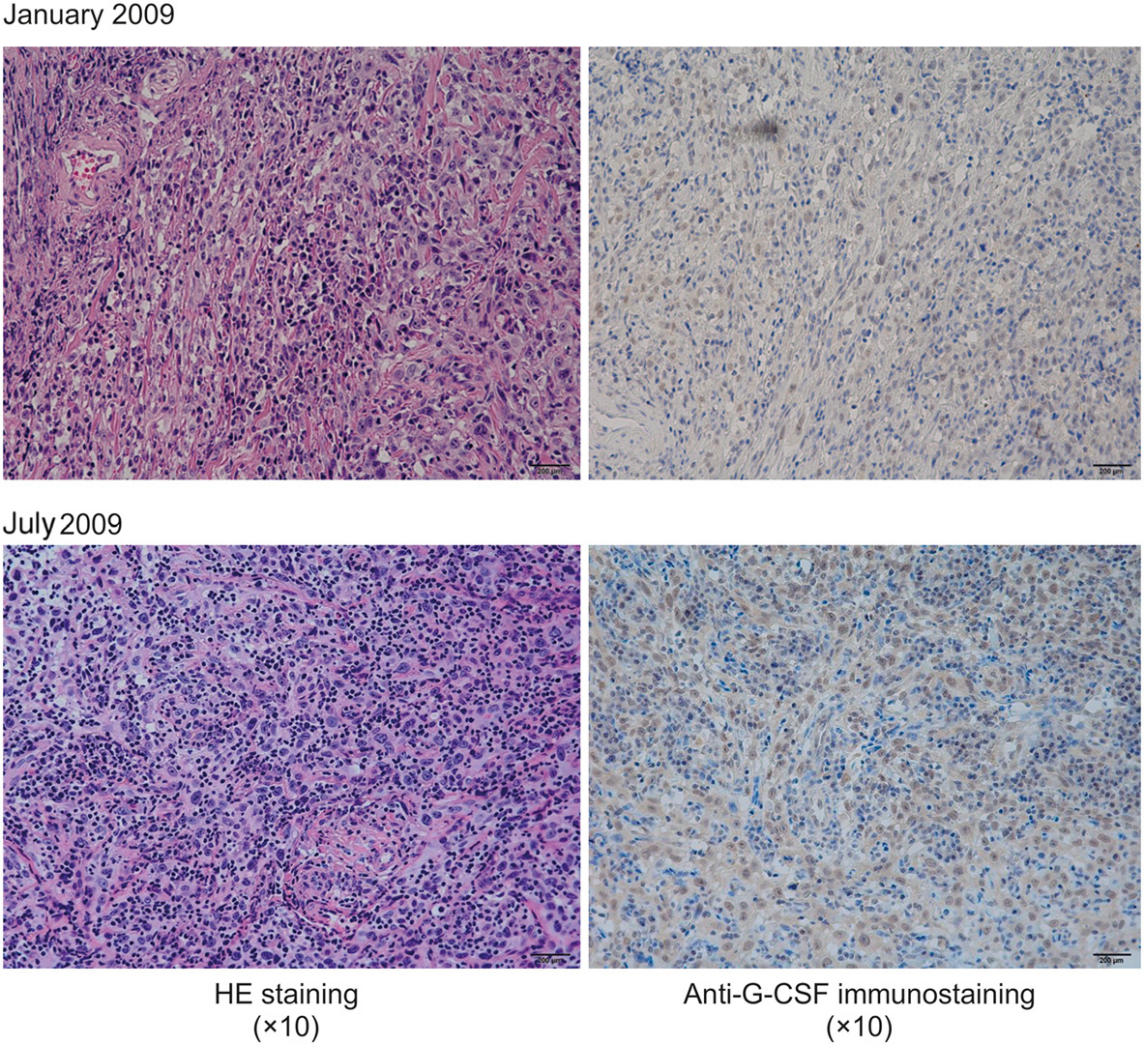
G-CSF-positive cells were more pronounced in the biopsy tissue specimen obtained in July 2009 than in the surgical specimen obtained in January 2009.

Pathological examination on relapse revealed well-differentiated SCC. Immunostaining of the resected specimen in January 2009 confirmed the presence of G-CSF-positive tumor cells. Staining of biopsy tissue in July of the same year revealed the G-CSF-positive tumor cells to be more pronounced (Figure 8).

## Discussion

It is known that solid malignancies are rarely accompanied by leukocytosis and other leukemoid symptoms, the mechanism of which is attributed to the production of G-CSF by the tumor cells themselves [1,2]. Inflammation, infection, and leukemia are all possible causes of marked leukocytosis. However, there are also malignant tumors that manifest as elevated WBC count that could not be explained, as illustrated in a case study by Hughes et al. in 1950 [3]. In Japan the first case of a G-CSF-producing tumor in the lung was reported by Asano et al. in 1977 [4]. In terms of site of onset, G-CSF-producing tumors are most often reported in patients with lung cancer [4,5], with other described cases including cancer of the stomach [6], pancreas [7], skin [8], kidneys [9], and uterine cervix [10,11]. However, there are relatively few studies on G-CSF-producing tumors occurring in the head and neck, including the upper jaw, gums, thyroid, tongue, and parotid glands [12-14].

Diagnosis of G-CSF-producing tumors previously required the use of cumbersome methods to demonstrate G-CSF production by tumor cells. Such methods included the *in vitro* colony formation assay, measurement of G-CSF activity after transplanting tumors into nude mice [4], and detection of G-CSF mRNA in tumor cells [15]. Currently, however, diagnosis is relatively simple, because G-CSF production in tumor cells can be demonstrated by measuring the serum G-CSF levels with enzyme immunoassay (EIA), or by immunohistochemical staining with rh-G-CSF antibodies [15].

Asano et al. proposed the following 4 criteria for diagnosing G-CSF-producing tumors: (1) marked granulocytosis, mainly consisting of mature neutrophils, (2) elevated serum and urine G-CSF levels, (3) normalization of granulocyte count and G-CSF level following tumor removal, and (4) demonstration of increased G-CSF in the tumor [4]. The patient in the present study exhibited an increased WBC count, up to 32 × 10^3^/ml with neutrophil count of 93 %, and an elevated serum G-CSF level of 250 pg/ml (normal G-CSF range, <18.1 pg/ml). G-CSF-positive tumor cells were identified using immunohistochemical staining. Thus, the patient fulfilled 3 of the 4 above-mentioned criteria, although normalization of granulocyte count and G-CSF level following tumor removal could not be determined. However, comparison of the G-CSF-positive tumor cells in an immunohistochemically stained specimen, surgically removed in January 2009, with those of the biopsy tissue specimen obtained in July of the same year, indicated a marked increase in the number of positive cells of the latter specimen. This finding, combined with the onset of leukocytosis after surgery, suggested a marked proliferation of the patient's G-CSF-producing tumor cells after resection of the tumor, leading to a diagnosis of G-CSF-producing SCC of the lower gum.

G-CSF-producing tumors are considered to have a poor prognosis, due to the effects of G-CSF on proliferating tumor cells and enhancement of metastasis. G-CSF may therefore accelerate the clinical progression of the disease [16]. The patient in the present study also exhibited sudden systemic metastasis after treatment, accompanied by deterioration of her general condition.

G-CSF acts on the bone marrow to increase the WBC count, but does not directly trigger inflammatory responses, such as fever and elevated CRP. However, previous studies have also reported findings of inflammation, inferring that a separate cytokine is simultaneously produced within the G-CSF-producing tumor, in conjunction with the inflammatory response. A previous report speculated that interleukin 6 (IL-6) could be the cytokine responsible for these effects [17]. The report on G-CSF-producing tumors accompanied by inflammatory responses, such as fever and elevated CRP, revealed high levels of serum IL-6. Similarly, the patient in the present study also had a high serum IL-6 level of 32.5 pg/ml (normal range, <4.0 pg/ml), suggesting that IL-6 is involved in the onset of fever and elevated CRP. IL-6 has been reported to promote G-CSF production [18]. Therefore, it is possible that IL-6 produced by the tumor elicited the production of G-CSF. While intratumoral G-CSF can be locally diagnosed with immunohistochemical staining, no such established diagnostic method currently exists for IL-6. Demonstration of the existence of IL-6-producing tumor cells would therefore require cultivation of tumor cells and measurement of their IL-6 activity, and will be the focus of a future study. The findings of the present study indicate that in solid-malignancy patients who develop leukocytosis, elevated CRP, and fever, it is important to make an early differential diagnosis between inflammatory disease and G-CSF-producing tumors. Early diagnosis and determination of a therapeutic strategy are also desirable, so as to improve prognosis.

## Conclusion

The present paper summarized our experience in treating a patient with G-CSF-producing SCC of the lower gum. The patient was diagnosed with a G-CSF-producing tumor on the basis of leukocytosis, elevated serum G-CSF, and G-CSF-positive tumor cells, immunohistochemically detected by anti-G-CSF antibodies, as well as clinical findings.

## Consent

Written informed consent was obtained from the patient for publication of this case report and any accompanying images. A copy of the written consent can be furnished upon demand.

## Abbreviations

G-CSF: Granulocyte colony-stimulating factor; SCC: Squamous cell carcinoma; PET/CT: Positron emission tomography/computed tomography; CT: Computer tomography; WBC: White blood cell; BLM: Bleomycin; CDDP: Cisplatin; Gy: Gray; UFT: Tegafur-uracil; CDGP: Nedaplatin; DTX: Docetaxel; CRP: C-reactive protein; EIA: Enzyme immunoassay; IL-6: Interleukin 6; FDG: Flurodeoxyglucose.

## Competing interests

The authors declare that they have no competing interests.

## Authors’ contributions

AM designed research and performed research. TY, KN, RS, TK, and NS performed research. HH designed research and performed research. All authors read and approved the final manuscript.
